# Therapeutic Antibodies Targeting CSF1 Impede Macrophage Recruitment in a Xenograft Model of Tenosynovial Giant Cell Tumor

**DOI:** 10.1155/2010/174528

**Published:** 2010-10-13

**Authors:** Hongwei Cheng, Paul W. Clarkson, Dongxia Gao, Marina Pacheco, Yuzhuo Wang, Torsten O. Nielsen

**Affiliations:** ^1^Department of Pathology and Laboratory Medicine, The University of British Columbia, Vancouver, BC, Canada V5Z 1M9; ^2^British Columbia Cancer Agency, Vancouver, BC, Canada V5Z 1L3

## Abstract

Tenosynovial giant cell tumor is a neoplastic disease of joints that can cause severe morbidity. Recurrences are common following local therapy, and no effective medical therapy currently exists. Recent work has demonstrated that all cases overexpress macrophage colony-stimulating factor (CSF1), usually as a consequence of an activating gene translocation, resulting in an influx of macrophages that form the bulk of the tumor. New anti-CSF1 drugs have been developed; however there are no preclinical models suitable for evaluation of drug benefits in this disease. In this paper, we describe a novel renal subcapsular xenograft model of tenosynovial giant cell tumor. Using this model, we demonstrate that an anti-CSF1 monoclonal antibody significantly inhibits host macrophage infiltration into this tumor. The results from this model support clinical trials of equivalent humanized agents and anti-CSF1R small molecule drugs in cases of tenosynovial giant cell tumor refractory to conventional local therapy.

## 1. Introduction

Tenosynovial giant cell tumor (TGCT) affects young adults, and can occur as a localized soft tissue neoplasm in the synovial lining of tendon sheaths (commonly known as giant cell tumor of tendon sheath) or in the lining of synovial joints, where it is commonly known as pigmented villonodular synovitis (PVNS) [1]. PVNS is locally aggressive, with the capacity to invade surrounding soft tissues and bone, erode the articular cartilage on the surface of the joint, and eventually cause significant morbidity through the development of secondary arthritis. Malignant progression of PVNS is uncommon but does occur [2]. In spite of treatment with total synovectomy, and even with adjuvant radiation therapy, PVNS has a high recurrence rate [1]. 

In most cases of tenosynovial giant cell tumor, a disease-specific *COL6A3-CSF1* translocation is detectable in a distinct subpopulation of tumor cells. The presence of high levels of CSF1 expression, recruiting a large body of macrophages to the tumor site, appears to be a consistent feature in all forms of this disease [3, 4]. Thus, aberrant CSF1 signaling plays a critical role in tumor development and progression in PVNS/TGCT, which may therefore represent the ideal index disease to test the therapeutic value of CSF1 inhibitors. The value of testing this strategy is of immediate clinical relevance because of the current lack of effective medical therapies for this disease. 

Imatinib is known to inhibit the macrophage colony-stimulating factor receptor (CSF1R), as evidenced in a recent dramatic report where imatinib treatment led to a complete response in one case of advanced recurrent PVNS [5]. However, subsequent studies have only demonstrated stable disease (Blay; personal communication). Recruitment of macrophages to tumor sites and regulation of their functional specialization through CSF1 promote growth and metastasis in many tumor types [6], and the development of agents blocking CSF1 signaling has therefore become an active focus of research [7, 8]. Given that imatinib is not a particularly strong inhibitor of CSF1R [9], it is possible that other agents, including investigational new drugs specifically designed to block CSF1 signaling, might be better choices. 

The immediate problem in designing preclinical studies for PVNS/TGCT therapies is a lack of suitable experimental models. As might be expected in a lesion where >90% of tumor cells are host macrophages, this disease cannot easily be modeled *in vitro*, and no cell lines currently exist. Furthermore, as with most tumors that grow slowly, standard subcutaneous xenografts have very poor take rates. In this study, we establish a new model for PVNS/TGCT by transplanting primary human tumor samples under the renal capsule of NOD SCID mice. We then develop an assay for recruitment of host macrophages into the implanted human tumor tissues, allowing us to test the effectiveness of imatinib and of antihuman CSF1 antibodies to block macrophage recruitment in this model system.

## 2. Materials and Methods

### 2.1. Primary Tumor Tissue and Clinical Information

Eight patients diagnosed with PVNS/TGCT requiring surgical excision at Vancouver General Hospital were consented for this experimental study. Volunteers were treated in accordance with the Canadian Tri-Council Policy Statement on the ethical conduct for research involving humans, and these studies were reviewed and approved by the BC Cancer Agency Research Ethics Board. Median patient age was 39.5 years, ranging from 20 to 50 years. Clinical data for these patients is summarized in Table 1.

### 2.2. Renal Subcapsular Xenotransplantation

Approximately 1 cc of fresh tumor tissue was obtained from each patient, and fragments of 3 × 2 × 1 mm were grafted under the kidney capsule of 20 to 36 nonobese diabetic/severe combined immunodeficiency (NOD SCID) mice. These methods are described in detail in previous published work and have a high rate of successful engraftment [10, 11]. 

 Animal care and experiments were carried out in accordance with the guidelines of the Canadian Council on Animal Care and were approved by the Animal Care Committee of The University of British Columbia. Meloxicam was administered subcutaneously prior to the surgery to provide long-term pain relief. Animals were weighed and anaesthetized with Ketamine/Xylazine (100 and 10 mg/kg resp., 0.1 ml/25 g body weight, intraperitoneal injection). Bupivacaine was administered as local anaesthetic at the injection site. An incision of approximately 2.5 cm was made along the midline of the skin in the back of the mouse. With the animal lying on its side, a small incision was made in the body wall with length slightly longer than the long axis of the kidney. The kidney was then exteriorized by applying pressure on its other side using the forefinger and thumb. The exteriorized kidney rested on the body wall. A fine pair of forceps was used to gently pinch and lift the capsule from the parenchyma of the kidney so that a 2–4 mm incision could be made in the capsule with fine spring-loaded scissors. A pocket was created between the kidney and the parenchyma by blunt dissection. Great care was taken not to damage the kidney parenchyma and thus prevent bleeding. The graft was transferred to the surface of the kidney using a blunt scalpel. The cut edge of the kidney capsule was lifted with a pair of fine forceps and the graft was inserted into the pocket under the capsule using a polished glass pipette. Once the grafting procedure was completed, the kidney was gently eased back into the body cavity; and the incision in body wall (muscle layers) was closed with 4–0 sutures; the edges of the back skin will be aligned and closed with the aid of suture. Buprenorphine was injected subcutaneously at 0.05–0.10 mg/kg (0.1 ml/25 g body weight) at the time mice show signs of recovery from anaesthesia.

### 2.3. Experimental Design

Two weeks after graft implantation, NOD SCID mice were randomly divided into treatment and control groups. For CSF1 antibody treatment, the mouse antihuman CSF1 monoclonal antibody 5H4 (ATCC accession HB10027), specific for human dimeric CSF1 and not cross-reactive with mouse CSF1 [12], was kindly provided by Novartis Institutes for Biomedical Research (Emeryville, CA, USA). 5H4 antibody was administered via intraperitoneal injection at 10 mg/kg, once per week. Imatinib was purchased from LC Laboratories (Woburn, MA, USA) and was given via gavage at 100 mg/kg, once per day, in five of eight cases where enough mice and tissue were available for this additional experimental arm. In one case, imatinib was administered at a concentration of 200 mg/kg. Aqueous vehicle (phosphate buffered saline = PBS) was used as control, given intraperitoneally once weekly. After two weeks of treatment, all mice were sacrificed in a CO_2_ chamber; both kidneys with their tumor grafts were removed, fixed in 10% neutral buffered formalin, and paraffin-embedded for histology, immunochemistry, and fluorescence in situ hybridization (FISH) studies. To evaluate longer-term 5H4 treatment effects, we also completed one experiment using 7-weeks treatment.

### 2.4. Paraffin FISH

Fluorescent in situ hybridization (FISH) analyses for *CSF1* gene disruptions were performed using break-apart hybridization probes, using the same protocol as previously described in [3]. For negative cases, a minimum of 500 nuclei were assessed in the primary tumor specimen.

### 2.5. Histopathology and Immunohistochemistry

H&E staining was performed for histopathologic evaluation using standard protocols. For immunohistochemistry, antigen retrieval was performed in 0.1 M citrate buffer (pH 6.0) in a steamer. Endogenous peroxidase activity was blocked with 3% hydrogen peroxide, and nonspecific protein binding was blocked with serum-free Protein Block (Dako, Carpenteria, CA). Rat antimouse macrophage F4/80 monoclonal antibody (Invitrogen Corp, Carlsbad, CA) was applied at 1 : 50 dilution and incubated overnight at 4°C. Biotinylated antirat secondary antibody (Dako, Carpenteria, CA) at 1 : 300 dilution was labeled with horseradish peroxidase-conjugated streptavidin (Vector Labs, Burlingame, CA) and visualized using Nova Red Substrate Kit (Vector Labs). Mouse antihuman Ki67 monoclonal antibody SP6 (Dako, Carpenteria, CA) at 1: 50 dilution and human macrophage CD163 (Novocastra, Newcastle, UK) at 1 : 100 dilution were applied by using the M.O.M. Kit (Vector Labs) according to the manufacturer's recommendations. Goat polyclonal antihuman CSF1 (GeneTex, Irvine CA) at 1 : 10 dilution was evaluated using the Vectastain Elite ABC goat Kit (Vector Labs), with the DAB chromogen (Dako) applied for 10 minutes at room temperature.

### 2.6. Immunohistochemical Scoring and Statistical Analysis

Immunohistochemical staining results were scored for the percentage of positive cells in a section from the middle of the whole grafted tumor area, except for human Ki67 (which was scored for the absolute count of positive nuclei per high power microscope field of xenograft tissue). Because a thin inflammatory layer usually exists surrounding the implanted tumor tissues as a surgical reaction, this outer layer was excluded from scoring for assayed markers including F4/80. All scoring work was done by a pathologist who was blinded to all drug treatment and sample identifiers; codes were only broken after all scores were finalized, and the scoring and data analysis were handled by separate researchers. Murine macrophages were identified by F4/80 staining, and the host macrophage infiltration index was defined as the number of F4/80 positive cells, divided by the total number of cells in the examined whole sections from the xenografted tumor. Results are expressed as percentages, and experimental error is reported corresponding to the 95% confidence intervals of the observed medians. The significance of treatment effects compared to PBS control was evaluated within each treatment group (5H4 versus PBS and imatinib versus PBS) using pairwise Wilcoxon rank sum tests. Foldchanges in macrophage infiltration rate are reported on aggregate as the mean fold-change across all mice within each treatment group. 95% confidence intervals of the mean foldchange were estimated from bias-corrected accelerated bootstrap calculations using 1000 replicates [13].

## 3. Results

### 3.1. A Renal Subcapsular Xenograft Model of Pigmented Villonodular Synovitis/Tenosynovial Giant Cell Tumor

For all cases, tenosynovial giant cell tumor xenografts implanted under the renal capsule of NOD SCID mice yielded viable human tumor tissue at sacrifice (at times ranging from one to six months). Tumors did not grow appreciably in size; the fraction of cells positive for human Ki67 was less than 1% and was not affected by drug treatment. To assess the representativeness of the PVNS/TGCT renal subcapsular xenografts, we examined side by side the harvested graft tissues and their corresponding primary tumor specimens, comparing their histomorphological features, *CSF1* translocation status, and CSF1 protein expression patterns.

**Figure 1:**
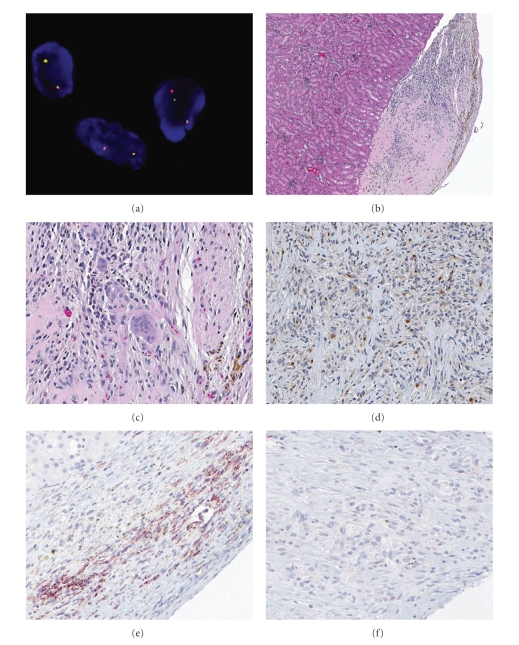
Renal subcapsular implant model of tensosynovial giant cell tumor. (a) Fluorescence in situ hybridization on xenograft using red and green probes flanking the *CSF1* locus, showing split probes in the rightmost cell, consistent with translocation of CSF1 sequences (objective magnification 100x). ((b)-(c)) H&E histology of the xenograft: (b) host kidney on the left and tumor on the right (objective magnification 10x); (c) centre of the xenograft (objective magnification 40x). (d) Immunohistochemistry for CSF1 in xenograft tissue (objective magnification 20x). ((e)-(f)) F4/80 immunohistochemistry (20x objective magnification), showing host macrophage infiltration in PVNS xenografts: (e) PBS control, (f) mice treated with 5H4 anti-CSF1 antibody.

The *CSF1 *translocation (Figure 1(a)) was identifiable in only three out of the eight case specimens recruited in this study, a finding not unexpected considering that the *CSF1* translocation can only be detected in about 60% of cases and even then in only a small minority of tumor cells (about 2%) [3]. Histologically, all examined PVNS/TGCT tumor grafts were very similar to their primary tumor counterparts (Figures 1(b) and 1(c)). However, whereas a mix of CD163+ human and F4/80+ murine macrophages present in the grafts two weeks after implantation, by four weeks murine host F4/80+ macrophages had replaced most of the human macrophages, and syncytial giant cells were rarely present. CSF1 expression exhibited an overall diffuse pattern in both tumor grafts and primary tumor tissues (Figure 1(d)), very similar to the CSF1 expression patterns previously described in [3, 4]. Tumors were stable to the lifespan of the NOD/SCID host (6 months) but subclones could not be passaged into a new generation.

### 3.2. An Anti-CSF1 Monoclonal Antibody Significantly Reduces Host Macrophage Infiltration into PVNS/TGCT Xenografts

**Figure 2:**
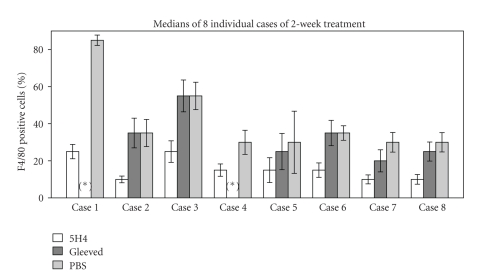
Effect of CSF1 inhibitors on macrophage infiltration in PVNS xenografts. (a) Data from experiments using eight individual patient tumors (host macrophages counted after 2 weeks of the indicated treatments). Error bars represent the 95% confidence interval of the observed median. In case number eight, the dose of imatinib was doubled to 200 mg/kg but showed similar effects. (*) denotes cases where imatinib was not given.

Human CSF1 is known to crossreact with and stimulate murine macrophages [8]. 5H4 is a mouse monoclonal antibody that specifically neutralizes human CSF1. In short-term *in vitro* culture of primary PVNS tumor tissue, 5H4 did not show any cytotoxic effects (data not shown). When administered by the intraperitoneal route to mice bearing PVNS xenografts, 5H4 significantly blocked murine host macrophage recruitment to the tumor site (Figures 1(e) and 1(f)). Effects of 5H4 on xenograft series derived from eight individual patients are shown in Figure 2, in comparison to PBS and imatinib. As scored by pathologists blinded to treatment group, 5H4 decreased the macrophage infiltration for all cases in 2-week treatment experiments, by an average of 2.7-fold in comparison with vehicle control (PBS) (95% confidence interval 2.3–3.0). In a single experiment where treatment was continued out to 7 weeks, the fraction of host macrophages among cells in the xenograft was 60% (95% CI 53%–67%) in the PBS arm as compared to 30% in the 5H4 arm (95% CI 24%–36%).

### 3.3. Imatinib Blocks Macrophage Infiltration to a Lesser Extent Than 5H4 in This Renal Subcapsular Implant Model

Since imatinib functions as a CSF1R inhibitor [9] and was highly effective in a PVNS clinical case report [5], we used it as a comparative control in this study when sufficient numbers of xenograft-bearing mice were available. We tested five patients' tissues at 100 mg/kg/day imatinib, a dose reported to effectively suppress metastases in breast cancer xenograft models [14] and one at 200 mg/kg/day (the maximum tolerable dose in short-term treatment [15]). Across all six experiments, imatinib decreased host macrophage infiltration by 1.1-fold relative to PBS control (95% confidence interval 1.0–1.3), an effect which was not statistically significant (Figure 2). Relative to 5H4, imatinib showed a lesser effect on reducing macrophage recruitment at these doses in this model system. Imatinib had no significant effect on the number of human Ki67-positive cells relative to controls (data not shown).

## 4. Discussion

Grafting human primary tumors beneath the renal capsule in NOD SCID mice is a recently developed method for the establishment of xenograft models. Relative to the low engraftment rate seen with subcutaneous implantation (typically 20–40% for aggressive carcinomas) [16], renal subcapsular implantation shows a high tumor take rate (possibly due to its high tissue perfusion environment) and is especially advantageous for establishing models of low-grade, slow-growing tumors [10, 11]. In our PVNS models, the estimated engraftment rate of grossly evident, microscopically confirmed viable tumor tissue was over 95%. Harvested grafts maintained characteristic features of the original tumors with respect to tumor morphology, *CSF1* translocation status, CSF1, and macrophage CD163 expression, making this the first PVNS model of practical use for preclinical therapeutic investigation. Although the implanted tumors did not grow appreciably in net size, we could assess the infiltration of F4/80+ host murine macrophages. In experiments using other tumor types which do not produce CSF1 (synovial sarcoma, clear cell sarcoma, and myxoid liposarcoma), no host F4/80+ macrophage infiltrates were seen (Cheng and Nielsen, unpublished observations), supporting that their presence in PVNS xenografts indeed occurs as a consequence of tumor CSF1 expression rather than as a reaction to the implantation procedure. This model can be used to test the effects of a new generation of drug agents designed to block CSF1-recruited macrophage infiltration. 

Tumor-derived CSF1 overexpression is a common finding in many types of human neoplasms [17, 18]. In conjunction with well-recognized roles in stimulating macrophage survival, proliferation, and differentiation [19], CSF1 appears to direct macrophages to adopt an M2 phenotype [20]. This macrophage subtype is involved in immune regulation, wound healing, and secondarily in tumor promotion, rather than in active phagocytic antipathogen immune responses. Intratumoral macrophage infiltration is seen in clinical and experimental studies of many tumor types and correlates with poor tumor prognosis, for example, in cancers of breast, prostate, ovary, and smooth muscle [20–23]. The mechanisms by which tumor associated macrophages are thought to promote tumor growth include roles in supporting angiogenesis, secretion of tumor growth factors, suppression of immunosurveillance, and enhancement of tumor metastasis [6, 24]. Thus accumulating evidence indicates a potentially important role of CSF1 signaling in cancer biology, and many anti-CSF1 approaches are currently being developed. For example, CSF1 antibody, antisense oligonucleotide, and *CSF1* small interfering RNA strategies have all demonstrated tumor suppression capabilities to various degrees in other disease and model systems [7, 8]. 

Since CSF1-activating translocation-driven macrophage recruitment is fundamental to the pathophysiology of PVNS [3, 4], this neoplasm plausibly represents an index disease model for assessing the value of anti-CSF1 therapeutics. In study, we tested the effect of 5H4, a mouse monoclonal antihuman CSF1-neutralizing antibody, for its ability to inhibit macrophage recruitment in our PVNS xenograft model. Based on the anti-CSF1 receptor activity of imatinib [9, 14, 25, 26] and a recent publication showing imatinib's therapeutic effects in one PVNS case [5], we chose imatinib as a positive control in our experiments. Relative to 5H4, imatinib exhibited a lesser macrophage blockade effect in the animal model at the doses employed (100 and 200 mg/kg). The target in this model system is host murine CSF1R, rather than human CSF1R, which could result in an underestimation of the benefit of imatinib; of note, recent studies have nevertheless proven that imatinib does inhibit phosphorylation of murine CSF1R [14].

## 5. Conclusions

This study introduces and characterizes a PVNS model which is suitable for anti-CSF1 therapeutic studies. We were able to demonstrate that anti-CSF1 therapies impede macrophage recruitment, supporting their potential value as therapeutic agents in this locally aggressive disease of joints for which no established, effective drug therapy currently exists.

## Figures and Tables

**Table 1:** Clinical information on the eight PVNS/TGCT cases used in this study.

Case ID	Presentation	Location	Size (cm)	Recurrent	CSF1 translocation
1	Diffuse	Knee	7	Yes	+
2	Diffuse	Knee	5	No	−
3	Nodular	Knee	6	No	+
4	Nodular	Foot	4	No	−
5	Nodular	Finger	1.9	No	+
6	Diffuse	Knee	10	No	−
7	Nodular	Knee	2	No	−
8	Diffuse	Hip	6	No	n/a

## Abbreviations

5H4: Murine antihuman CSF1 monoclonal antibody clone 5H4
CSF1: Macrophage colony-stimulating factor
FISH: Fluorescence in situ hybridization
NOD SCID: Nonobese diabetic severe combined immunodeficient
PVNS: Pigmented villonodular synovitis
TGCT: Tenosynovial giant cell tumor.
